# Understanding pregnancy planning in a low-income country setting: validation of the London measure of unplanned pregnancy in Malawi

**DOI:** 10.1186/1471-2393-13-200

**Published:** 2013-11-05

**Authors:** Jennifer Hall, Geraldine Barrett, Nicholas Mbwana, Andrew Copas, Address Malata, Judith Stephenson

**Affiliations:** 1 UCL Institute for Global Health, 30 Guilford Street, London, UK; 2 School of Health Sciences and Social Care, Brunel University, Uxbridge, UK; 3 MaiMwana Project, Mchinji, Malawi; 4 Department of Infection & Population Health, UCL Institute of Epidemiology and Health Care, London, UK; 5 Kamuzu College of Nursing, University of Malawi, Lilongwe, Malawi; 6 Research Department of Reproductive Health, UCL Institute for Women’s Health, London, UK

**Keywords:** Pregnancy intention validation Malawi measure unplanned

## Abstract

**Background:**

The London Measure of Unplanned Pregnancy (LMUP) is a new and psychometrically valid measure of pregnancy intention that was developed in the United Kingdom. An improved understanding of pregnancy intention in low-income countries, where unintended pregnancies are common and maternal and neonatal deaths are high, is necessary to inform policies to address the unmet need for family planning. To this end this research aimed to validate the LMUP for use in the Chichewa language in Malawi.

**Methods:**

Three Chichewa speakers translated the LMUP and one translation was agreed which was back-translated and pre-tested on five pregnant women using cognitive interviews. The measure was field tested with pregnant women who were recruited at antenatal clinics and data were analysed using classical test theory and hypothesis testing.

**Results:**

125 women aged 15–43 (median 23), with parities of 1–8 (median 2) completed the Chichewa LMUP. There were no missing data. The full range of LMUP scores was captured. In terms of reliability, the scale was internally consistent (Cronbach’s alpha = 0.78) and test-retest data from 70 women showed good stability (weighted Kappa 0.80). In terms of validity, hypothesis testing confirmed that unmarried women (p = 0.003), women who had four or more children alive (p = 0.0051) and women who were below 20 or over 29 (p = 0.0115) were all more likely to have unintended pregnancies. Principal component analysis showed that five of the six items loaded onto one factor, with a further item borderline. A sensitivity analysis to assess the effect of the removal of the weakest item of the scale showed slightly improved performance but as the LMUP was not significantly adversely affected by its inclusion we recommend retaining the six-item score.

**Conclusion:**

The Chichewa LMUP is a valid and reliable measure of pregnancy intention in Malawi and can now be used in research and/or surveillance. This is the first validation of this tool in a low-income country, helping to demonstrate that the concept of pregnancy planning is applicable in such a setting. Use of the Chichewa LMUP can enhance our understanding of pregnancy intention in Malawi, giving insight into the family planning services that are required to better meet women’s needs and save lives.

## Background

80 million women in developing countries experienced an unintended pregnancy in 2012 resulting in an estimated 30 million unplanned births, 40 million abortions and 10 million miscarriages [1]. 63 million of these unintended pregnancies are at least in part a consequence of the fact that 222 million women worldwide have an unmet need for family planning [1].

Whilst all pregnancies expose women to some risk, unintended pregnancies expose women to these risks unnecessarily and without them making the decision to take on these potential risks for the benefit of having a child. In developing countries pregnancy can carry an extremely high risk of morbidity and mortality; in 2012 approximately 291,000 women in developing countries died from pregnancy-related causes. That 104,000 of these women will not have wanted to become pregnant in the first place makes this even more of a tragedy. Were the unmet need for family planning fully met it is calculated that 79 000 maternal deaths could be prevented each year [1]. The majority of these would be in sub-Saharan Africa where there are high levels of both unmet need and maternal mortality.

In order to meet the unmet need for family planning we need to develop a better understanding of women’s pregnancy intentions and behaviours. Most current estimates of the levels of unplanned pregnancy in developing countries are derived from questions used in the Demographic and Health Survey (DHS). The standard DHS question asks “At the time you became pregnant, did you want to become pregnant then, did you want to wait until later, or did you not want to have any (more) children at all?” Whilst this has provided useful information, there has been increasing discussion of the limitations of these types of questions and of the need to develop a more sophisticated method for measuring this complex construct [2-9].

The London Measure of Unplanned Pregnancy (LMUP) is a new tool for measuring the degree of pregnancy intention of a current or recent pregnancy [3]. It was developed in the United Kingdom (UK) [4] and has subsequently been formally translated and validated in India and the United States of America [10,11] with unvalidated translations in use elsewhere. By asking six questions, each scored zero, one or two, the LMUP scores pregnancy intention on a continuous scale from zero to 12 with each increase in score representing an increase in the degree of pregnancy intention [3]. By scaling intention in this way the dichotomisation of pregnancies into planned and unplanned is avoided and women are able to express ambivalence. The questions cover contraceptive use, timing, intention, desire for a baby, discussion with the partner and pre-conception preparation (see http://www.lmup.co.uk for the full English version).

The LMUP has the potential to be a useful tool for understanding pregnancy intention in a range of settings but must be translated and validated prior to use outside of the context in which it was developed. The aim of this study was to translate and validate the LMUP for use in the Chichewa language in Malawi using classical test theory.

Malawi is a low-income country ranking 170 out of 187 countries in the Human Development Index. It has a high maternal mortality of 460 per 100 000 live births [12] and 26% of married women have an unmet need for family planning leading to 45% of pregnancies being reported as unplanned [13]. These factors make it an ideal candidate to be the first location for a validation of the LMUP in a low-income country. The research was conducted in Mchinji District, a Chichewa speaking district in the central region of Malawi. Mchinji has an estimated population of 530,218 people with 23% (121,950) being women of childbearing age (Mchinji socio-economic profile 2012, unpublished).

## Methods

The LMUP was originally designed for self-completion. Given the low levels of literacy in Mchinji District [13] this was not felt to be a viable option. The LMUP was therefore adapted for interviewer-administration along the same lines as the Indian validation [11].

The interviewer-administered English LMUP was sent to three native Chichewa speakers (two female, one male, all involved in health research) who each independently translated it into Chichewa. All translators were given a short briefing on the purpose and background of the LMUP prior to conducting the translation. The three translations were reviewed by JH and the differences were discussed at a consensus meeting of the three translators plus a locally trained nurse-midwife and health researcher. The agreed translation produced by this meeting was sent for back-translation to a native English speaker who spoke Chichewa fluently as a second language. This person was only broadly aware of the purpose of the LMUP.

Following back-translation the Chichewa LMUP was pre-tested using cognitive interviewing techniques. The aim of these interviews was to gauge the ease with which women understood the questions, to check the translation and to assess the acceptability of the questions. Pregnant women were recruited for these interviews from Mchinji District Hospital (MDH) antenatal clinic.

The final version of the Chichewa LMUP was field-tested at three antenatal clinics in Mchinji District: MDH, Kochilira Community Hospital and Ludzi Community Hospital. Three women living in these areas who had previously worked with our organisation were trained to conduct the interviews. All pregnant women aged 15 or over attending any one of these clinics in the week of 8th October 2012 were invited to participate. Given the accepted guidance for an appropriate sample size for the validation of a questionnaire, 100 was selected as the target total sample size with at least 50 completing the re-test [14].

The interviewer verbally explained the purpose of the research to the potential participant with the aid of a written information sheet that the participant retained. All women completed the six LMUP questions, and a short set of demographic and obstetric history questions, and were invited to return to the same antenatal clinic on any day the following week to complete the re-test. They were offered 500 Malawian Kwacha (£1/US$1.52/€1.15) to cover their transport costs if they returned. The women were given a unique identification number on a card that they were advised to bring with them when they returned. This number was used to link the test and re-test data as no personal identifiable data was collected.

Respondent’s answers were inputted directly onto password protected Personal Digital Assistants (PDAs) during the interview to maximise the safety of the data. Pendragon software was used to design the questionnaires and to control what data can be entered, reducing the risk of errors during data entry. Data was transferred directly to an Excel spreadsheet on a laptop via USB, eliminating transcription errors. All data were anonymous but were stored in encrypted files.

### Analysis of psychometric properties

The analysis was conducted in STATA version 12 using a Classical Test Theory-based approach to facilitate comparison with the original UK study and previous validations [10,11].

In addition to the feedback from the cognitive interviews, acceptability was assessed by examining missing data rates with lower levels of missing data indicating greater acceptability [15]. To assess item discrimination the item-endorsement values were checked to ensure that no item had an endorsement of greater than 80% [16]. The distribution of total scores was considered to evaluate the targeting of the scale and ensure that the full range of scores was captured.

To assess reliability, internal consistency was evaluated by calculating the Cronbach’s α statistic using the standard cut off point of 0.7 [17]. In addition all item-rest correlations were examined with a minimum correlation of 0.20 considered acceptable [16]. Test-retest stability was assessed using the weighted κ with a score above 0.60 considered to be substantial [18]. The simple Pearson’s correlation between scores at the two administrations was also assessed; 0.60 was the cut-point for acceptable correlation as was used in the validation in India [11].

As there is no agreed 'gold standard’ for the measurement of pregnancy intention it is not possible to assess the concurrent criterion validity of the LMUP by comparing it to this.

Construct validity was examined using two methods: hypothesis testing and principal component analysis. Hypotheses were generated based on the literature on pregnancy intention and hypotheses used in previous LMUP validations adapted to suit the Malawian context [3,10,11]. Given the non-parametric distribution of pregnancy intention scores the Wilcoxon Rank-Sum (Mann Whitney U) test was used to test the three main hypotheses that: pregnancies will be reported as more unplanned (i.e. LMUP score will be lower) in women with a four or more live children; women who are unmarried; and women aged under 20 or over 30. Principal component analysis (PCA) was used to evaluate the internal structure of the LMUP. The scale would be considered valid if all items load onto one component with an Eigenvalue larger than one (i.e. are measuring the same construct) [19]. Our findings led to us conducting a sensitivity analysis to determine the effect of removing the first question (contraception use) on the validity of the scale.

## Ethical approval

The University College London Research Ethics Committee and the College of Medicine Research Ethics Committee at the University of Malawi granted ethical approval for this study. Approval to conduct the research in Mchinji District’s antenatal clinics was given by the District Medical Officer. Written informed consent to participate was taken with thumbprints used if women were illiterate.

## Results

### Pre-testing

Cognitive interviews were conducted on five pregnant women attending the Mchinji District Hospital antenatal clinic. The women were aged 17 – 38 (median 20) and four of the five women were married. They had between three and nine years of education; had had between zero and six previous pregnancies and were between six and nine months pregnant.

In general the women reported that the instructions were easy to follow and the questions easy to understand. The main change that was made during the cognitive interviews was on the first question; contraceptive use in the month they became pregnant. General knowledge of contraception seemed variable and this impacted on the answers given to the first question. For example, two women reported not using contraception but on further probing they were (one had had a tubal ligation, one was using a natural method of family planning). Probing around this issue revealed that the women only seemed to think of methods such as pills, injections or condoms and did not think beyond these. Four of the five understood that family planning was a way of 'stopping pregnancy’ but it seemed that the women were interpreting the Chichewa word 'zolerera’ too narrowly. There was not a better word available and so it was decided that we would preface the question with some additional information to help the respondents. The text that was added was:

'This question asks about contraception. This might include condoms, pills, injections, implants, coils, vasectomy, female sterilisation or any other method aimed at delaying pregnancy’.

The second change that was made was to alter the options available for question six, pre-pregnancy preparations, to include the more contextually relevant option 'saved money for healthcare.’ This is not applicable in the UK but is relevant in the Malawian context and indeed was included in the Indian validation. Discussion with local women and midwives indicated that the smoking and alcohol responses were unlikely to be relevant in this context however the decision was made to include them in the field test and base their inclusion or exclusion on the data collected.

### Field-test: women’s characteristics

Data were collected from one hundred and twenty five women, surpassing the target of 100. Women were aged from 15–43 (median 23, mean 24.5) and had between zero and seven live children (median 1). Eighty percent of the women were married and the majority (69.6%) had primary education only (see Table 1).

**Table 1:** Characteristics of women completing the London Measure of Unplanned Pregnancy (LMUP) field test and re-test compared to the averages for Mchinji District and Malawi as a whole where available in the Demographic and Health Survey (DHS)

**Socio-demographic characteristics**	**LMUP field test n = 125**	**LMUP retest n = 70**	**LMUP non-retest n = 55**	**Comparison of retest and non-retest groups**	**Mchinji DHS 2010 data**^**i**^	**Malawi DHS 2010 data**^**i**^
**Age**						
Mean (sd)	24.4 (5.9)	25.03 (6.1)	24.4 (6.3)	P = 0.3120		
Median	23	25	22			
Range	15 – 43	15 - 41	16 - 43			
**Age group**	**N (%)**	**N (%)**	**N (%)**			
15-19	28 (22.4)	14 (20)	14 (25.5)			
20-24	41 (32.8)	20 (28.6)	21 (38.2)			
25-29	28 (22.4)	19 (27.1)	9 (16.4)			
30-34	19 (15.2)	13 (18.6)	6 (10.9)			
35-39	7 (5.6)	3 (4.3)	4 (7.3)			
≥40	2 (1.6)	1 (1.4)	1 (1.8)			
**Children**						
0	39 (31.2)	21 (30)	18 (32.7)	P = 0.2549		
1	35 (28.0)	16 (22.9)	19 (24.6)			
2	23 (18.4)	15 (21.4)	8 (14.5)			
3	10 (8.0)	5 (7.1)	5 (9.9)			
≥4	18 (14.4)	13 (18.6)	14 (25.5)			
**Marital status**						
Married	101 (80.8)	54 (77.1)	47 (85.5)	P = 0.242	68%	81.3%
Unmarried	24 (19.2)	16 (22.9)	8 (14.5)		32%	19.7%^ii^
**Education**						
None	6 (4.8)	3 (4.3)	3 (5.5)	P = 0.978	18.2%	15.2%
Primary	87 (69.6)	48 (68.6)	39 (70.1)		64%	64.8%
Secondary	29 (23.2)	17 (24.3)	12 (21.8)		17.8%	18.1%
Tertiary	3 (2.4)	2 (2.9)	1 (1.8)		0%	1.8%
**Partner’s occupation**	Missing data for 5		Missing data for 5			
Unemployed/Student	10 (8.3)	5 (7.1)	5 (10)	P = 0.076	7.9%	18%
Agriculture/Casual labour	53 (44.2)	37 (52.9)	16 (32)		72.4%	82%
Employed/Business man	57 (47.5)	28 (40)	29 (58)		19.7%	

^i^DHS data refers to all women all aged 15–49 and not only to pregnant women ^ii^Never married rather than currently unmarried.

### Field test: psychometric properties

**Figure 1:**
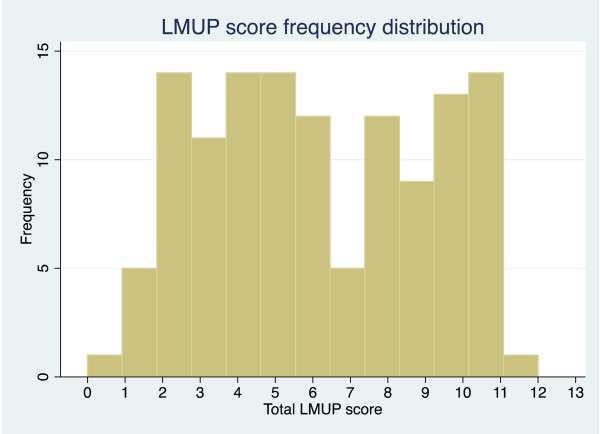
Distribution of Chichewa London Measure of Unplanned Pregnancy (LMUP) scores.

There were no missing data and no question had a response with more than 80% endorsement. The full range of LMUP scores from zero to twelve was captured in the field test (see Figure 1). The median score was 6.

The Cronbach’s α for the whole scale was 0.78. Item-rest correlations were above or around 0.7 for questions two to five, was borderline for question six (0.16) and was low for question one (0.05) (see Table 2).

**Table 2:** Principal component analysis of Chichewa London Measure of Unplanned Pregnancy

		**Component 1 (Eigenvalue = 3.1)**	**Component 2 (Eigenvalue = 1.0)**
**Items**	**Item-rest correlations**	**Item loadings**	**Item loadings**
1 - Contraception	0.05	-0.04	*0.99*
2 - Timing	0.69	*0.48*	0.07
3 - Intention	0.79	*0.51*	-0.02
4 - Desire	0.74	*0.50*	0.06
5 - Partner	0.72	*0.48*	-0.03
6 - Preparation	0.16	*0.14*	0.07

74 women returned for the re-test but due to interviewer error data were only available on 70. The women who returned for the re-test were not significantly different from those who did not return in terms of age, parity, number of live children, marital status, education or partner’s occupation (see Table 1). The average test-retest interval was 7 days (range 5–10 days). The median difference in the scores at test and re-test was zero (mean -0.2). The weighted κ statistic was 0.799 and the Pearson correlation coefficient was 0.801 showing good stability.

**Figure 2:**
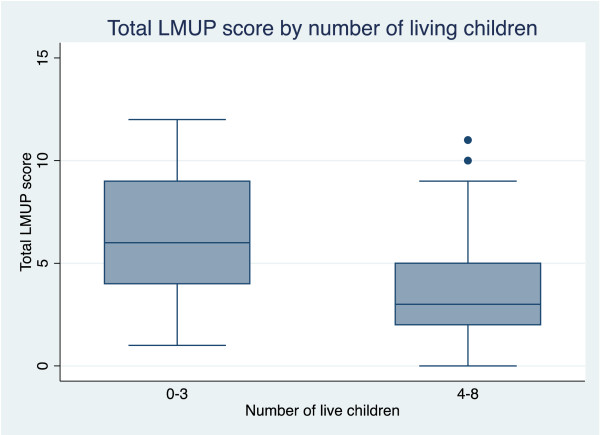
Box plot showing median and inter- quartile range of London Measure of Unplanned Pregnancy (LMUP) score by number of living children.

**Figure 3:**
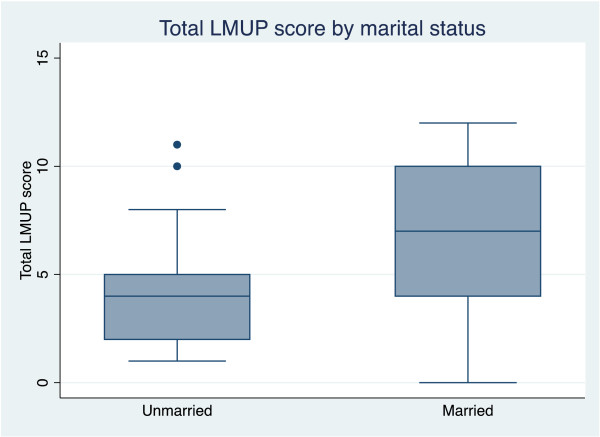
Box plot showing median and inter- quartile range of London Measure of Unplanned Pregnancy (LMUP) score by marital status.

**Figure 4:**
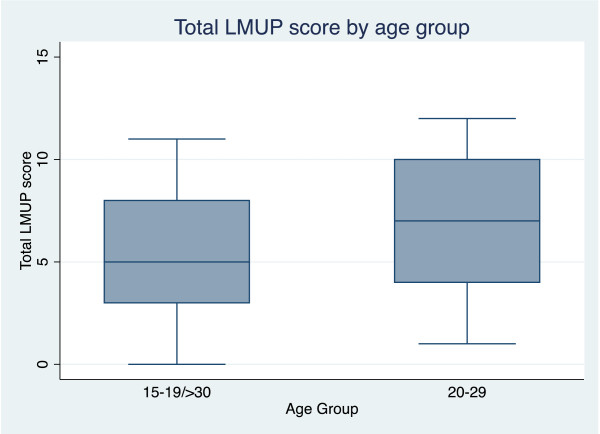
Box plot showing median and inter- quartile range of London Measure of Unplanned Pregnancy (LMUP) score by age group.

Hypothesis testing confirmed that women who already had four or more children alive (p = 0.0051), unmarried women (p = 0.003), and women who were below 20 or over 29 (p = 0.0115) were all more likely to report their pregnancies as more unintended (see Figure 2, Figure 3, Figure 4).

Principal component analysis confirmed that five items clearly measured one construct loading onto one component with an Eigenvalue of 3.1. A second component was of borderline significance with an Eigenvalue of 1.00 and mainly represented the question on contraception (loading of 0.99), in keeping with the lower item-rest correlation (see Table 2).

### Field test: sensitivity analysis

The LMUP was re-analysed without the question on contraception use. This reduced the LMUP scores to zero to 10 and gave a median score of 4 for our data. Cronbach’s α increased from 0.78 to 0.83 and all items loaded on to one component with an eigenvalue of 3.10. All hypothesis tests remained statistically significant (data not shown).

### Finalisation of the Chichewa LMUP

The responses to the question on pre-pregnancy preparation were inspected to determine which options should remain in the final version of the Chichewa LMUP. No respondents reporting cutting down on smoking (99.8% of women in Mchinji do not use any form of tobacco [13]) and only one woman reported cutting down on alcohol. These items were therefore removed from the final version of the Chichewa LMUP.

## Discussion

The validation of the Chichewa LMUP using classical test theory shows that the Chichewa LMUP meets the pre-set criteria for acceptability, endorsement, targeting, internal consistency, reliability and construct validity by hypothesis testing. The original English LMUP has now been translated and validated into five other languages in high-, middle- and low-income countries. Although analysis by classical test theory shows slightly weaker performance by all translations than the original, they remain acceptable (see Table 3).

**Table 3:** Comparison of results of classical test theory analysis for validation of the original London Measure of Unplanned Pregnancy and its translations

	**Internal consistency Cronbach’s α**	**Eigenvalues of principal component analysis components**	**Test retest weighted κ**
UK	0.92	4.33	0.97 and 0.86
USA - English	0.78	2.9	0.72
USA - Spanish	0.84	3.4	0.77
India - Kannada	0.76	2.66 and 1.05	0.43
India - Tamil	0.71		
Malawi	0.78	3.1 and 1.00	0.80

The only slight deviation from the pre-set criteria for the Chichewa LMUP was on the principal component analysis. Here all items were expected to load onto one component with an Eigenvalue larger than one thus demonstrating that all components are measuring the same construct. In actual fact they loaded onto two components with an Eigenvalue larger than one, although the second component had an eigenvalue of 1.00 making it very borderline. The same thing was found in the Indian validation and it was noted that the 'second component … mainly represented item one [contraception] (loading of 0.78)’ [11]. In the Chichewa LMUP this was also true with the second component almost entirely representing the question on contraception (loading of 0.99). The Mokken analysis conducted in the USA validation indicated that the contraception question 'was not contributing greatly to the scale [but] the scale was still strong with the inclusion of this item’ [10].

In the original LMUP not using contraception was more strongly associated with intention to become pregnant than it appears to be in any of the subsequent translations. In the Malawian setting this might be explained by the fact that there is a high unmet need for family planning i.e. 27% of married women who do not want another pregnancy in the next two years are not using any form of family planning [13]. In this context the relationship between not using contraception and wanting to get pregnant is diluted. Similar factors may also be at play in the Indian setting and in the USA study that was conducted in low-income women. We recommend retaining the question on contraception in the Malawian setting for several reasons. Firstly, the scale is not compromised by its retention, secondly if the LMUP is used over time we may see this item becoming more relevant as unmet need for family planning falls and, finally, to enable easier comparison with LMUP use elsewhere.

### Limitations

There are three main limitations to this study. Firstly, in Malawi abortion is illegal so we were not able to test the LMUP in women who we knew were and were not planning to continue the pregnancy to term. Despite this the Chichewa LMUP could be used in women following induced or spontaneous abortion as it was developed and validated with abortion as an outcome of pregnancy in the original UK development [3]. Secondly, we were only able to conduct a test-retest analysis during pregnancy. Subsequent work is underway that will allow a postpartum re-test analysis to be conducted. Finally, we recruited women from antenatal clinics meaning that we missed women who do not attend for antenatal care. Although in Mchinji District over 90% of women receive antenatal care from a skilled attendant at least once during their pregnancy [13] the 10% of women who do not attend are likely to be significantly different from those who do in many ways. This might account for why the women in this study tended to have higher levels of education and of partner employment than was expected from the district level data in the DHS as seen in Table 1.

## Conclusion

The Chichewa LMUP is a valid and reliable measure of pregnancy intention in women who speak Chichewa and is now an available tool for research and surveillance in Malawi. It is the first time the LMUP has been formally validated in a low-income country and in so doing it helps to demonstrate that the concept of pregnancy planning is applicable in these settings. The Chichewa LMUP represents a methodological advance on the DHS-style pregnancy intention questions, particularly by allowing a more nuanced picture of pregnancy intention and planning, and can be used for a range of research questions pertaining to pregnancy intention such as enhancing understanding of pregnancy planning behaviour or investigating relationships between pregnancy intention and maternal and neonatal health. This should lead to insights for the provision of family planning programmes to aid Malawi in designing programmes to meet the unmet need for family planning and reduce maternal and child deaths.

## Abbreviations

DHS: Demographic and Health Survey; LMUP: London Measure of Unplanned Pregnancy; MDH: Mchinji District Hospital; PDA: Personal digital assistant; PCA: Principal component analysis; UK: United Kingdom; USA: United States of America.

## Competing interests

The authors declare that they have no competing interests.

## Authors’ contributions

JH and GB designed the study with the support of JS and AM. JH and NM trained the fieldworkers and oversaw the data collection process. JH analysed the data with advice and input from GB and AC. JH wrote the first draft of the article, which all authors contributed to. All authors have read and approved the final manuscript.

## Pre-publication history

The pre-publication history for this paper can be accessed here:

http://www.biomedcentral.com/1471-2393/13/200/prepub
